# Targeting pulmonary vascular endothelial cells for the treatment of respiratory diseases

**DOI:** 10.3389/fphar.2022.983816

**Published:** 2022-08-30

**Authors:** Yi-Xuan Li, Hong-Bo Wang, Jing Li, Jian-Bo Jin, Jing-Bo Hu, Chun-Lin Yang

**Affiliations:** ^1^ School of Materials Science and Chemical Engineering, Ningbo University, Ningbo, China; ^2^ Department of Pharmacy, Yuyao People’s Hospital, Yuyao, China

**Keywords:** respiratory diseases, vascular endothelial cells, drug delivery systems, acute lung injury, pneumonia

## Abstract

Pulmonary vascular endothelial cells (VECs) are the main damaged cells in the pathogenesis of various respiratory diseases and they mediate the development and regulation of the diseases. Effective intervention targeting pulmonary VECs is of great significance for the treatment of respiratory diseases. A variety of cell markers are expressed on the surface of VECs, some of which can be specifically combined with the drugs or carriers modified by corresponding ligands such as ICAM-1, PECAM-1, and P-selectin, to achieve effective delivery of drugs in lung tissues. In addition, the great endothelial surface area of the pulmonary vessels, the “first pass effect” of venous blood in lung tissues, and the high volume and relatively slow blood perfusion rate of pulmonary capillaries further promote the drug distribution in lung tissues. This review summarizes the representative markers at the onset of respiratory diseases, drug delivery systems designed to target these markers and their therapeutic effects.

## Introduction

Vascular endothelial cells (VECs) play an important regulatory role in many pathological processes (Shuvaev et al., 2011). VECs, lined on the inner surface of blood vessel wall as an osmotic barrier, can regulate the balance of blood vessel tension, blood vessel wall damage repair and coagulation-fibrinolysis by secreting active substance (Howard et al., 2014a). The functional integrity of VECs is crucial to the support for the improvement of diseases. More and more attention has been paid to the effective delivery of drugs to damaged VECs, starting with the improvement of vascular endothelium and achieving effective treatment of diseases.

VECs can play a part in a variety of biological functions. In the meantime, it is also a better target site. Most drugs are not selective for VECs. Many receptors specifically expressed on VECs have a high affinity to the corresponding ligand modified drugs or biomaterials to realize the active target transport (Brenner et al., 2015). The expression of specific receptors on VECs was different under different physiological and pathological conditions. For example, the decreased expression of angiotensin-converting enzyme and increased expression of intercellular adhesion molecule-1 (ICAM-1) were observed in inflammatory and other pathological states. Nevertheless, the constant expression of platelet endothelial cell adhesion molecule-1 (PECAM-1) were observed both in physiological and pathological states. Pulmonary vascular endothelial cells are divided into pulmonary macro-(PAEC) and microvascular (PMVEC) endothelial cells, which have different phenotypes. Compared with PAECs, PMVECs have a faster growth rate and can continue to grow in the absence of serum. Moreover, PMVECs also have stronger restrictive barriers. In experiments using lectins to distinguish the two phenotypes, PAECs preferentially interact with lectins, which may affect their response to specific inflammation (King et al., 2004). Hence, understanding the expression of specific receptors on VECs is crucial for the design of VECs-targeting drug delivery systems for different purposes including prevention, diagnosis or treatment.

Compared with other tissues, the distribution of VECs-targeting drug delivery systems *via* intravenous administration has a significant binding advantage in lung tissues. In addition to specific receptors expressed on VECs, there are three reasons for advantages of pulmonary VECs-targeting therapy: 1) The greater endothelial surface area of pulmonary vessels (approximately one-third of the total endothelial surface); 2) The “first pass effect” of venous blood in lung tissues. Venous blood from the right ventricle flows first through the capillary, then through the left ventricle, where it flows to the endothelium; 3) The pulmonary artery is the first major capillary after intravenous administration of the drug, and its high volume, relatively slow blood perfusion rate promotes the combination of targeted drugs and specific receptors expressed on VECs in the bloodstream. Considering that the pulmonary VECs are involved in the regulation of various respiratory diseases, the precise treatment of respiratory diseases can be achieved through the VECs-targeting drug delivery systems.

**FIGURE 1:**
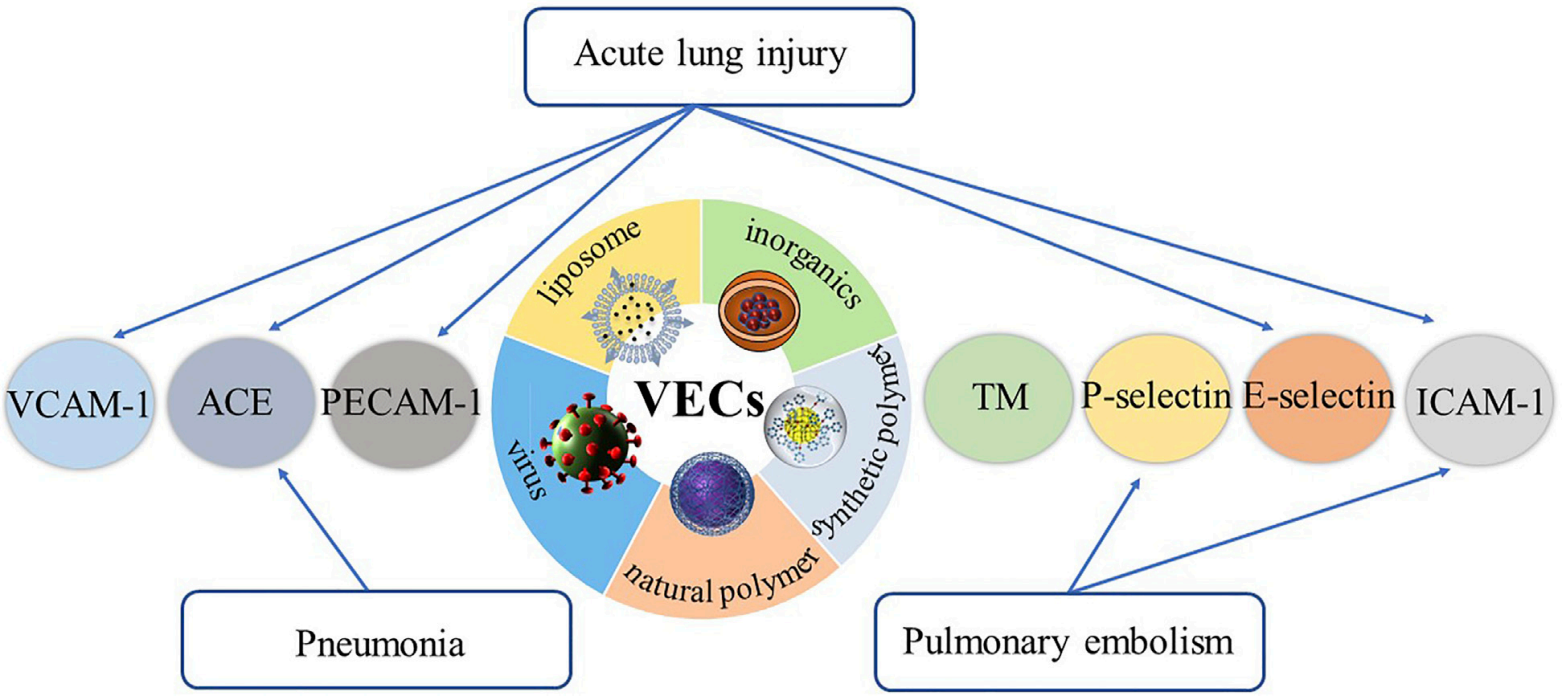
The representative specific receptors expressed on VECs at the onset of respiratory diseases, and drug delivery systems designed to target these receptors.

This review summarizes the representative specific receptors expressed on VECs at the onset of respiratory diseases, drug delivery systems designed to target these receptors, their therapeutic effects and future research directions (Figure 1, Table 1).

**TABLE 1:** Summary of drug-delivery nanoparticles targeting lung endothelial cells.

Particle type	Target	Nanocarrier	Disease	Consequence	*In vitro* (cells)	*In vivo* (mouse models)	References
Polymer	ICAM-1	Anti-ICAM NCs		Reduce sICAM-1 by decreasing cell-surface ICAM-1 during endocytosis	HUVECs		Manthe and Muro (2017)
Polymer	ICAM-1		Pneumonia	Reduce the presence of nanoparticles in the system organ, significantly increase the distribution of nanoparticles in the lungs		BALB/c mice	Anselmo et al. (2015)
Liposomes	ICAM-1	Anti-ICAM-1/SV/NLCs	Acute lung injury	Increase biocompatibility, reduce side effects, reduce nonspecific diffusion	EAhy926	The male Balb/c mice	Li et al. (2017)
Liposomes	ICAM-1	Anti-ICAM/tPA	Pulmonary embolism	Effectively dissolve fibrin micro embolism in rat lung	REN, HUVECs	Sprague-Dawley rats	Murciano et al. (2003)
Polymer	PECAM	Anti-PECAM/NCs		Carrier endocytosis was achieved without affecting the endothelial barrier	HUVECs, REN		Garnacho et al. (2008)
Peptide	PECAM	Anti-PECAM svFv/TM M388L	Oxidative stress vascular inflammation and thrombosis	Good endothelial targeting properties and antioxidant activity	MS1, REN	C57BL6J mice	Carnemolla et al. (2013)
Polymer	VCAM-1/PECAM-1/ICAM-1	PS-NPs	Inflammation	Obvious competitive distribution advantage	H5V	C57BL/6J mice	Papademetriou et al. (2014)
Polysaccharide	P-selectin	Fu/DOX	Breast cancer	Higher cytotoxicity to cancer cells, lower side effects	MDA-MB-231, MDA-MB-468, MCF-12A		Cumashi et al. (2007)
Polysaccharide	P-selectin	rt-PA-Fuco-NPs	Thrombus	Enhance P-selectin interaction *in vitro* and the efficiency of rt-PA *in vivo*		SCID mice	Juenet et al. (2018)
BSA	E- selectin	Esbp-modified BSANPs	Acute lung injury	Better cell uptake and blood compatibility, good inhibition of inflammatory reaction	HUVECs	Kunming mice	Liu et al. (2019)
Liposomes	ICAM-1	NCL/Pro/Ang	Acute lung injury	Significantly reduce the level of proinflammatory factors, with good distribution *in vivo*	EAs	BALB/c mice	Jiang et al. (2019)
Liposomes	ACE	Anti-ACE Ab/tPA	Pulmonary embolism	distinct pulmonary distribution		Sprague-Dawley rats	Muzykantov et al. (1996)

## Representative receptors on pulmonary vascular endothelial cells

On the basis of the high affinity with drugs or carriers, the ideal receptors on pulmonary VECs should meet the following criteria: 1) Accessibility. The targeting receptors are extensively expressed on the surface of VECs, effectively exposed to the vascular lumen and with enough space to effectively bind to drugs or carriers modified by corresponding ligands (van den Hoven et al., 2013). 2) Safety. The interaction between receptors and drugs or carriers may lead to the disordered intracellular signal transduction, and abscission or internalization of epitope. What’s more, it may not only interfere with VECs, but also affect the biological function of some targets, which causes potential side effects, including complement and phagocyte activation, allergic reactions, and inflammation that can damage the liver, kidney, and lung. Consequently, these potential side effects should be fully considered in the design of drug delivery systems, and the necessary *in vivo* safety monitoring should be carried out (Szebeni et al., 2012). 3) Specificity. The specific receptors expressed on VECs are of great significance to realize the exclusive targeting of VECs. At the same time, it should be noted that non-endothelial cells which express the same receptors with VECs can be ignored if they don’t contact with blood circulation. 4) Accurate subcellular localization of drugs. An ideal VECs-targeting drug delivery system, based on the effective binding with endothelial cell receptors, should be able to further achieve appropriate localization at subcellular level according to therapeutic objectives. For example, antithrombotic drugs should be maintained on the cell surface as much as possible; enzyme replacement therapy drugs should be delivered to lysosomes. The representative VECs-targeting receptors are discussed below.

### Constitutively expressed transmembrane glycoproteins

VECs-targeting constitutively expressed transmembrane glycoproteins, represented by angiotensin converting enzyme and thrombomodulin, extensively expressed on the surface of VECs. More importantly, their expression in pulmonary VECs is higher than that in other parts of the body.

### Angiotensin-converting enzyme

Angiotensin-converting enzyme (ACE) is a transmembrane glycoprotein expressed on the surface of VECs. It has numerous biological functions, which not only catalyze the conversion of Angiotensinogen I (Ang I) into Angiotensinogen II (Ang II), but also inactivate bradykinin, promote vasoconstriction and increase blood pressure. The percentage of endothelial cells expressing ACE on pulmonary VECs was close to 100%, while the expression on the other endothelial cells was less than 15% (Danilov et al., 2001). This high expression on pulmonary VECs significantly enhances the lung distribution advantage of the ACE-targeting drug delivery system (Metzger et al., 2011). ACE targeting was first reported in the late 1980s. Danilov et al. diagnosed the pulmonary vessels using an indium-111 labelled monoclonal antibody to ACE (Danilov et al., 1989). At the same time, Muzykantov et al. (1989) evaluated the increased lung accumulation of glucose oxidase after the conjugation with monoclonal antibody. Since then, a series of ACE targeting studies have been reported. Miller et al. (2005) used the ACE-specific FAB9B9 monoclonal antibody to modify the nitric oxide synthase adenovirus (AdeNOS) to increase the lung distribution and eNOS expression, and effectively reduce the blood pressure level in stroke-prone spontaneously hypertensive rats, which showed the effective application of ACE targeting in lung gene delivery. Balyasnikova et al. (2006) developed a series of monoclonal antibodies, recognized epitopes on the N- and C-domains of catalytically active ACE. These monoclonal antibodies had highly efficient and selective lung accumulation, which provided the potential for targeting drugs/genes to the pulmonary vasculature in different lung diseases (Balyasnikova et al., 2006). In the meantime, ACE specific monoclonal antibody 9B9 modified catalase has been reported to improve ischemia-reperfusion-induced lung injury and lung transplantation in rats.

The discovery of ACE-2 as the receptor for SARS-CoV-2 implicated the renin-angiotensin-aldosterone system in acute respiratory distress syndrome and respiratory failure in patients with COVID-19 (Rossi et al., 2020). ACE-2 is highly expressed in endothelial cells of lungs. Upon binding to ACE-2, SARS-CoV-2 activates the transmembrane serine protease-2 highly expressed in the lungs. Through fusion of its envelope with the cell membrane, the virus penetrates into the cells. The entrance of virus into cells can be prevented by SARS convalescent sera or by TMPRSS2 inhibitors, such as camostat and nafamostat mesylate. Targeting inhibition of ACE-2 is a potential therapeutic strategy for SARS-CoV-2 penetrating into pulmonary cells.

Many reports show that ACE has a good prospect in lung targeting therapy, but its safety use needs to be further studied. The target binding of ACE probably leaded to its inhibition or abscission, which then reduces the production of Ang II, increases bradykinin levels, promotes vasodilation, and lowers blood pressure (Nowak et al., 2013). This secondary effect can cause serious harm in patients who suffer from acute hypotension and angioedema. It is of great significance to understand the patient’s condition and monitor the potential side effects *in vivo* in order to promote the clinical application of ACE-targeting therapy. In addition, reactive oxygen species (ROS) and inflammatory cytokines can inhibit the expression of ACE, which may negatively affect ACE targeting efficiency (Muzykantov, 2005). To some extent, ACE-mediated lung endothelial targeting strategy may be more important in disease diagnosis and prevention than in treatment.

### Thrombomodulin

As a single-chain transmembrane glycoprotein on endothelial cells, TM was highly expressed on pulmonary VECs. As early as Trubetskoy et al. (1992) constructed TM-targeted n-terminal modified poly-l-lysine to deliver genes efficiently *in vitro* and *in vivo* on pulmonary VECs. TM consisted of 557 amino acids has anticoagulant and anti-inflammatory properties, which plays an important role in maintaining vascular smooth, but also can inhibit the adhesion between white blood cells and activated endothelial cells (Ito et al., 2019). Drożdż et al. (2018) reported that TM is a valuable marker of endothelial dysfunction in children with chronic kidney disease. But like ACE, TM’s biological function greatly limits its application in VECs-targeting strategy. TM has the biological function of promoting thrombin from procoagulant to anticoagulant, and it is an important intravascular thrombin inhibitor (Christofidou-Solomidou et al., 2002). However, the bind of targeting molecules to TM may cause TM blocking, inhibition or abscission, affect the anticoagulant activity of thrombin and increase the risk of thrombotic side effects. This potential side effect limits the clinical application of TM targeting, which is still in preclinical research (Shuvaev et al., 2007).

### Constitutively expressed cell adhesion molecules

Cell adhesion molecule (CAM) is a class of transmembrane glycoproteins belonging to Ig-superfamily (Ig-SF), which are also expressed on the surface of VECs in normal physiological state. Under normal physiological conditions, CAM does not have the characteristic of up-expression of ACE and TM in pulmonary VECs, but has a more homogeneous expression on the blood vessel endothelium *in vivo*. However, due to the distribution advantage of targeting drugs in the pulmonary endothelium during systemic circulation, CAM-mediated drug delivery systems have good lung distribution after intravenous administration (Papademetriou et al., 2014).

### Platelet-endothelial adhesion molecule-1

PECAM-1 is expressed at the molecular level of about (0.2–2) × 10^6^ per endothelial cell on average, and plays an important role in the integrity of endothelial cell connection (Lertkiatmongkol et al., 2016). PECAM-1 is also expressed in platelets and leukocytes, but its expression is much lower than that in endothelial cells, and therefore PECAM-1-targeting nanocarriers still has the advantage of endothelium competitive binding. PECAM-1 is abundant in physiological and pathological states, and it does not change with pathological state. This enables that PECAM-1-based nanocarriers can effectively target vascular endothelium under normal physiological or pathological conditions, thus making it of great significance for the prevention and treatment of related diseases. PECAM-1 is distributed throughout the body, so the distribution in the body is controlled by the particle size of the nanoparticles. The effect of flow on endocytosis by human endothelial cells of NC targeted by monoclonal antibodies Ab_62_ and Ab_37_ to distinct epitopes on the distal extracellular domain of PECAM was investigated by Han et al. The results showed that flow modulates endothelial endocytosis of Ab/NC mediated by PECAM-1 in an epitope-specific manner, *via* mechanisms involving complex and differential signaling pathways (Han et al., 2015). Therefore, when designing and constructing nanocarriers, attention should be paid to the influence of flow rate and other factors on drug efficacy.

PECAM-1 has the function of promoting the combination of inflammatory cell endothelium and infiltration into tissues, as well as internalizing the drugs or nanocarriers through CAM-mediated endocytosis. Therefore, PECAM-1 targeting intervention potentially regulates inflammation and other diseases. The molecular mechanism of endocytosis of PECAM-1-conjugated nanocarriers was investigated, and the results showed endothelial uptake of multivalent anti-PECAM complexes was associated with PECAM-1 phosphorylation (Garnacho et al., 2008). The PECAM-1 tyrosine 686 in cytoplasmic domain was critical in mediating RhoA activation and recruitment of EGFP-RhoA to anti-PECAM/nanocarriers binding sites at the plasmalemma, actin polymerization into phalloidin-positive stress fibers, and finally CAM endocytosis of anti-PECAM/nanocarriers (Garnacho et al., 2008). This discovery is of great help to the cellular uptake of nanocarriers. However, PECAM-1-mediated signal transduction is relatively complex including the regulation of platelet function and thrombosis (Gibbins, 2004). The comprehensive monitoring of PECAM-1 targeting security is of great significance to extend the targeting application of PECAM-1 and exert its endothelial targeting advantage.

Carnemolla et al. (2013) fused exogenous M388L mutant thrombomodulin (TM), resistant to oxidative inactivation, with the single-chain variable fragment (scFv) of mouse PECAM-1 antibody (anti-PECAM scFv/TM M388L). The results showed that anti-PECAM scFv/TM M388L had good endothelial targeting property and anti-thrombotic activity of TM under oxidative stress (Carnemolla et al., 2013). The paired antibodies directed to adjacent, yet distinct epitopes of PECAM stimulate each other’s binding to endothelial cells, thus improving targeting of therapeutic fusion proteins. PECAM antibodies-coated multivalent nanocarriers (Ab/NC) did mutually improve endothelial cell binding of Ab/NC coated by paired, but not “self” antibodies (Chacko et al., 2015). This “collaborative enhancement” effect was more pronounced for relatively large multivalent carriers versus free antibodies, providing a potential paradigm for optimizing endothelial cell-targeting nanocarriers. PECAM-1 antibodies modified liposomes with a particle size of 197.8 ± 4.5 nm were used to encapsulate antioxidant molecule EUK-134 for scavenging excessive reactive oxygen species (ROS) in the vascular endothelium of lung tissues (Howard et al., 2014b). Nucleoside modified mRNA-LNPs were coupled to antibodies specific for vascular cell adhesion molecule PECAM-1 (Abs). MRNA delivery and protein expression were approximately 200-fold and 25-fold elevated in the lung compared with their untargeted counterparts. The results demonstrated that LNP-mRNA targeting by antibody was independent of apolipoprotein E-mediated uptake pathway (Parhiz et al., 2018). This study provided the basis for the development of targeted delivery systems for mRNA therapy in lung diseases, including acute lung injury, pulmonary hypertension, etc.

### Inducible cell adhesion molecules

The expression of inducible cell adhesion molecules is very low on resting VECs, and significantly enhances on activated VECs after stimulation with proinflammatory factors, endotoxin, etc. In theory, inducible cell adhesion molecules are more suitable for the diagnosis and treatment of diseases.

### Intercellular adhesion molecule-1 (ICAM-1, CD54)

Under normal physiological conditions, ICAM-1 is expressed on endothelial cell lumen. ICAM-1 is also expressed in fibroblasts, epithelial cells and muscle cells, and its expression level was similar to that of ICAM-1 on vascular endothelium. However, since these non-endothelial cells do not come into contact with the blood circulation, ICAM-1-targeting delivery systems after intravenous infusion still have the advantage of competitive integration with pulmonary VECs. The expression of ICAM-1 was low in resting endothelial cells, while that was significantly increased by pro-inflammatory factors in inflammatory and pathological states (Muro et al., 2006). The uninhibited but enhanced expression of ICAM-1 in pathological state can improve the specific distribution of ICAM-1-targeting delivery systems in the lesion site (especially in the lung) and reduce the systemic side effects, which has great potential applications and important significance in the diagnosis and drug delivery of cancer, cardiovascular, lung, genetic, and other diseases, especially in the diagnosis and treatment of inflammation (Hsu et al., 2012).

The high expression of ICAM-1 participates in the adhesion and migration of leukocytes in blood vessels, and promotes the infiltration of inflammatory cells into and out of blood vessels; synchronously the activated leukocytes adhering to the endothelium further release reactive oxygen species and other pathogenic factors, which aggravate endothelial injury. In addition, Hopkins et al. (2004) reported that ICAM-1 also has the function of regulating some pathogens to invade tissues and pass through the blood-brain barrier. Therefore, compared with the possible hypotension side effects caused by ACE-targeting delivery, the binding of ICAM-1-targeting drugs or delivery systems to ICAM-1 will bring potential anti-inflammatory and other body protective secondary effects. Manthe and Muro (2017) have studied the potential secondary effects of ICAM-1-targeting nanocarriers, focusing on the effect of anti‐ICAM nanocarriers on endothelial release of soluble ICAM‐1, an inflammatory regulators. It was found that ICAM-1-targeting nanocarriers inhibited soluble ICAM‐1 release by mobilizing ICAM‐1 from the cell‐surface into intracellular vesicles. Therefore, this ICAM-1-targeting strategy provided a secondary benefit, in view of the upregulated ICAM-1 being closely related with numerous diseases. Clabl-conjugated poly (DL-lactate-co-glycolic acid) nanoparticles (ClABL-NP) were used to target ICAM-1 in lung epithelial cells. Compared with non-targeted NP, CLABL-NP showed specific binding to ICAM-1. Clabl-NPs also entered lung epithelial cells overexpressing ICAM-1 more rapidly. Drug release studies showed that ClABL-NP with high molecular weight PLGA exhibited sustained drug release dependent on pH (Chittasupho et al., 2009).

The internalization of ICAM-1-targeting delivery systems on endothelial cells may also promote its anti-inflammatory effect (Bhowmick et al., 2012). ICAM-1 antibody itself does not possess endothelium internalization, but ICAM-1 antibody-mediated vector can enter endothelium *via* the CAM-mediated endocytic pathway, which is different from the classical endocytic pathway such as phagocytosis and pinocytosis (Calderon et al., 2011). During CAM-mediated endocytosis, the ICAM-1 protein on the surface of VECs enters the cells together with the ICAM-1-targeting nanocarriers, then separates from nanocarriers within 1 h after endocytosis, and at last returns to the cell surface for subsequent internalization of nanocarriers. The transient endocytosis of ICAM-1 during CAM-mediated endocytosis does not affect the effective endocytosis of targeting nanocarriers. Even more importantly, internalization of ICAM-1 reduces the adhesion and exudation of inflammatory cells to a certain extent, which further exerts a potential anti-inflammatory effect (Muro et al., 2005).

**FIGURE 2:**
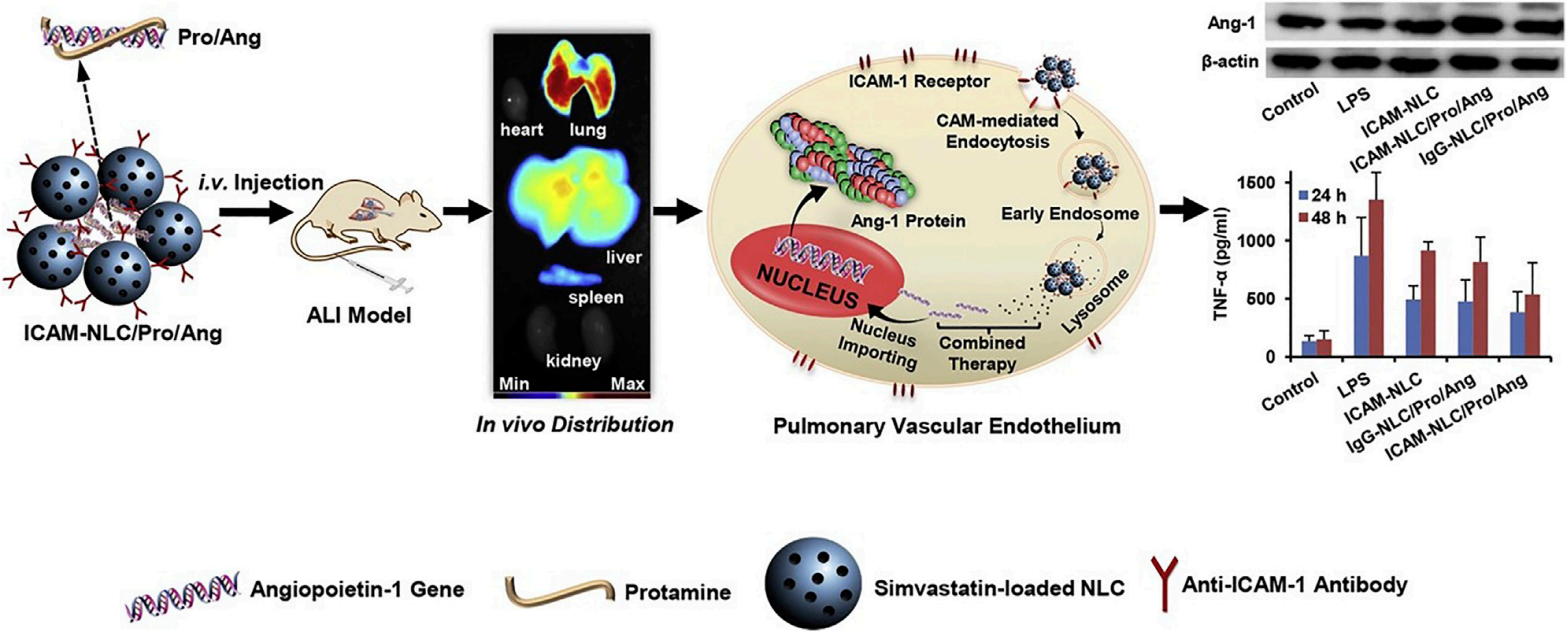
The ternary ICAM-NLC/Pro/Ang nanoparticles containing angiopoietin-1 gene, protamine and ICAM-NLC were prepared *via* charge interaction firstly. The ICAM-NLC/Pro/Ang exhibited ideal lung-targeted ability in lipopolysaccharide-induced ALI mice after i.v. administration, as well as the significant upregulation of Ang-1 protein in lung tissue. The ICAM-NLC/Pro/Ang realized an effective attenuation of pulmonary inflammation *via* co-delivery of Ang-1 gene and simvastatin to the injured lung. Collectively, the ICAM-NLC/Pro/Ang may represent a promising candidate favoring the clinical ALI therapy (Jiang et al., 2019). It was permitted.

Lung targeting strategies mediated by ICAM-1 have been widely reported (Papademetriou et al., 2013a). Anselmo et al. (2015) designed one “red blood cell delivery system modified by ICAM-1 monoclonal antibody” to avoid immune system clearance and enhanced the lung distribution. ICAM-1 antibodies-conjugated nanostructured lipid carriers encapsulating simvastatin (anti-ICAM-1/SV/NLCs) were prepared for the treatment of acute lung injury (ALI) (Li et al., 2017). The obtained anti-ICAM-1/SV/NLCs with a particle size of 354.7 ± 18.2 nm and polydispersity index of 0.229 ± 0.051 had good pulmonary distribution in lipopolysaccharide (LPS)-induced ALI mice. The anti-ICAM-1/SV/NLCs effectively ameliorated the ALI progresses, as reflected by inflammatory factors, pulmonary cells infiltration and pathologic changes. On this basis, angiopoietin-1 (Ang-1) gene, which regulated endothelial cells survival, vascular stabilization and angiogenesis, was combined with anti-ICAM-1/SV/NLCs (ICAM-NLC/Pro/Ang) for the treatment of ALI. The therapeutical effect of ICAM-NLC/Pro/Ang was significantly better than that of anti-ICAM-1/SV/NLCs. ICAM-NLC/Pro/Ang treatment upregulated expression of Ang-1 protein in lung tissues and further enhanced anti-inflammatory activities, which was associated with the effects of Ang-1 protein on endothelium structural integrity (Figure 2) (Jiang et al., 2019). Acid sphingomyelinase deficiency in type B Niemann-Pick disease results in lysosomal sphingomyelin storage, primarily affecting the lung, liver, and spleen. Nanocarriers (anti-ICAM/ASM NCs) targeting ICAM-1 increased lung uptake of recombinant enzymes. Lung delivery was optimized by different enzyme doses and nanocarrier concentrations, and the delivery activity and efficacy were evaluated. Anti-ICAM/ASM NCs switch the enzyme uptake mechanism from mannose-6-phosphate dependent clathrin mediated pathway to an ICAM-1 dependent endocytosis *via* the CAM pathway, resulting in enhanced lysosomal delivery (Garnacho et al., 2017). In the study of type B Niemann-Pick disease, Garnacho and Muro (2017) also enhanced the biodistribution and lysosomal delivery of NCs by targeting ICAM. Asthma is a heterogeneous airway inflammatory disease, and regulation of LFA-1 and/or ICAM-1 presents a promising and underappreciated novel therapeutic strategy (Hurrell et al., 2020).

Both ^125^I-labeled anti-ICAM and reporter enzyme (β-Gal) conjugated anti-ICAM showed good pulmonary endothelium accumulation. In addition, the tissue-type plasminogen activator (tPA) modified by ICAM-1 antibody effectively dissolved the fibrin micro embolism in the lung tissues of rats, reflecting the potential application of ICAM-1 targeting in the treatment of pulmonary embolism (Murciano et al., 2003).

### Vascular cell adhesion molecule-1

As a member of Ig-SF, Vascular cell adhesion molecule-1 (VCAM-1) is extensively expressed on the surface of endothelial cells for mediating leukocyte adhesion and metastasis. Its expression increases after being activated by pro-inflammatory factors, high glucose concentration, shear stress, endotoxin, etc. Naringenin, an anti-inflammatory citrus flavonoid, can be incorporated into VCAM-1 targeted lipid nanocarriers (V-Nar/ICG/LN) to improve the water solubility and bioavailability of naringenin. The studies *in vivo* showed a significant increase in V-Nar/ICG/LN accumulation in the heart and aorta compared with non-targeted lipid nanocarriers (Fuior et al., 2020). VCAM-1 targeted Fe_3_O_4_@SiO_2_ (FITC) nanoparticles, with highly efficient magnetic resonance imaging contrast properties, were able to target inflammatory endothelial cells. Nanoparticles typically had a diameter of 355 ± 37 nm, exhibited superparamagnetic behavior at room temperature, accumulated and targeted adhesion to inflammatory subunits of LPS-activated human umbilical vein endothelial cells (HUVECS-CS) (Yang et al., 2013). Therefore, it had high potential in vascular magnetic resonance imaging for clinical diagnosis of cardiovascular diseases, such as atherosclerosis and thrombosis.

VCAM-1 is also associated with the progression of immune disorders such as asthma, rheumatoid arthritis, transplant rejection, and cancer (Kong et al., 2018). It was reported that VCAM-1 was aberrantly expressed in tumor cells, and it could mediate distinct tumor-microenvironment interactions in leukocyte-rich microenvironments of the lungs to facilitate their metastasis. (Chen et al., 2011). The potent VCAM-1 inhibitor succinobucol-loaded nanoparticles were orally administrated and effectively inhibited the lung metastasis of breast cancer (Cao et al., 2015). The experiments in porcine reproductive and respiratory syndrome virus (PRRSV) natural infection and experimental infection of porcine lung injury model showed that the combined expression of VCAM-1 and IL-8 may be a biomarker of viral infection induced lung injury (Liu et al., 2015).

Nonetheless, the expression of VCAM-1 in activated VECs is not superior to that of PECAM-1 and ICAM-1, and therefore the single VCAM-1 targeting delivery strategy is not widespread. Therefore, the VCAM-1-based delivery strategy is commonly combined with other endothelial cell markers (McAteer et al., 2012). Papademetriou et al. (2014) designed polystyrene microspheres with single target, double targets, and triple targets for ICAM-1, PECAM-1, and VCAM-1. The results showed that PECAM-1/VCAM-1 double-targeting microspheres had stronger endothelial binding than that of single VCAM-1-targeting microspheres, and the PECAM-1-targeting microspheres had a significant competitive distribution advantage over single VCAM-1-targeting microspheres. Moreover, ICAM-1/PECAM-1/VCAM-1 triple-targeting microspheres showed a higher affinity to VECs than that of the double-targeting microspheres under inflammatory pathology, but a lower affinity to VECs under the physiology condition. The multi-targeting modification on nanocarrier could optimize its competitive distribution at the lesion site (Papademetriou et al., 2014). In addition, VCAM-1-targeting strategy is also used for detection. Bruckman et al. (2014) designed VCAM-1-targeting tobacco mosaic virus as magnetic resonance imaging contrast agent for atherosclerotic plaques. It should be noted that VCAM-1-targeting nanocarriers were ingested *via* a grid protein mediated pathway, so the size of nanocarriers should be less than 200 nm (Papademetriou et al., 2013b).

### P-selectin

P-selectin, belonging to the selectin family, is a type I single-chain transmembrane glycoprotein. It is mainly expressed in Weibel-Palade corpuscles of endothelial cells and alpha particles of platelets, and also expressed on the surface of plasma membrane under the stimulation of histamine, reactive oxygen species, complement and thrombin. The highly expressed P-selectin is involved in the adhesion between endothelial cells, platelets and white blood cells, and promotes the infiltration of white blood cells into tissues by promoting the rolling and adhesion of white blood cells on the surface of vascular endothelium. Fucoidan, a kind of sulfated polysaccharide from the ocean, is mainly found in the cell wall matrix of various brown algae, such as kelp, fucus, Undaria pinnatifida, etc. It has a variety of biological and pharmaceutical activities, including anti-cancer, anti-microbial, anti-virus, anti-inflammatory, and anti-coagulant activities (Cumashi et al., 2007). It was reported that fucoidan had a strong affinity towards P-selectin (Tong et al., 2018). Several fucoidan-based drug delivery systems had been developed for the treatment of tumor, acute kidney injury, bacterial infection, brain inflammatory, etc. (Manivasagan et al., 2019; Don et al., 2021; Lu et al., 2022).

Endothelial cell targeting, fucoidan-based micelles were fabricated to encapsulate antioxidant curcumin for the treatment of acute kidney injury (AKI) (Shu et al., 2021). The fucoidan-based micelles had good renal accumulation in AKI mouse models in comparison to that in healthy mice, which was associated with increased expression of P-selectin. Ferrante et al. (2009) constructed a P-selectin/VCAM-1 double-targeted tetrafluoromethane lipid microbubble contrast agent for arteriosclerosis plaque detection. This P-selectin/VCAM-1 double-targeted microbubble had higher adhesion efficiency than single-targeted microbubbles in the model flow chamber under high shear stress, showing its good potential in the diagnosis of pathological endothelium under the condition of high blood flow. Polysaccharide-poly (isobutyl cyanoacrylate) nanoparticles, rt-PA-Fuco-NPs, functionalized with fucoidan and loaded with rt-PA were designed to accumulate on the thrombus. In this study, they demonstrated that rt-PA-Fuco-NPs could effectively enhance P-selectin interaction *in vitro* and the efficiency of rt-PA *in vivo* (Juenet et al., 2018).

The expression of P-selectin in activated platelets weakens its endothelial targeting specificity to some extent. Therefore, it is of great significance to design P-selectin and other endothelial cell markers for synergetic endothelial targeting. P-selectin/VCAM-1 dual-targeted microparticles of iron oxide (DT-MPIO) was developed as a smart MRI probe to examine inflammatory activity in atherosclerosis (Chan et al., 2022). This DT-MPIO-enhanced MRI agent specifically targets high-risk vulnerable plaques, effectively differentiates the heterogeneity within the asymptomatic plaque population and quantitatively reports the inflammatory activity of local plaques in carotid artery (Chan et al., 2022).

### E-selectin

E-selectin is a member of the selectin family, with its molecular weight of 60 kDa. E-selectin is only expressed in endothelial cells stimulated and activated by inflammatory factors, reactive oxygen species, etc. (Bevilacqua et al., 1989). Therefore, E-selectin has a higher endothelial specificity than P-selectin. Furthermore, E-selectin is abundantly expressed in the vicinity of inflammation, cancer, and infection, and has become a good target for several therapeutic and medical imaging applications. E-selectin-targeted drug delivery systems were developed for the treatment of acute kidney injury, rheumatoid arthritis, spinal cord injury and cancer (Hu et al., 2017; Wang et al., 2019; Xu et al., 2020). In addition, E-selectin-targeted drug delivery systems were also prepared by using immunoconjugates and liposomes as carriers (Jubeli et al., 2012). E-selectin-binding peptide (Esbp)-modified bovine serum albumin (BSA) nanoparticles (BSANPs) were prepared to encapsulate dexamethasone for the treatment of ALI (Liu et al., 2019). Esbp-modified BSANPs could be preferentially internalized by TNF-α-induced HUVECs. Biodistribution results further demonstrated that Esbp-modified BSANPs had good accumulation in the lungs of ALI mice. Liposomes composed of 1,2-dipalmitoyl-sn-glycero-3-phosphocholine (DPPC), Cholesterol, and 1,2-Distearoyl-sn-glycero-3-phosphoethanolamine-N-[methoxy (polyethylene glycol)-2000]-maleimide (DSPE-PEG-Mal) were loaded with rapamycin and surface-modified E-selectin antibody. Liposomes were used for targeted delivery to E-selectin over-expressing TNF-α activated endothelial cells. The internalization of rapamycin loaded immune liposomes could inhibit the proliferation and migration of endothelial cells and the expression of inflammatory mediators (Gholizadeh et al., 2018).

Under the stimulation of pathogenic factors, the expression time of E-selectin lags behind that of P-selectin. Compared with PECAM-1 and activated ICAM-1, E-selectin has no advantage, and it can also be used in combination with other endothelial cell markers (Gunawan et al., 2011). It is noteworthy that the expression of E-selectin on endothelium is “transient,” and P-selectin has the same expression characteristics. Gunawan et al. (2011) stimulated endothelial cells through TNF-α or IL-1β. The results showed that the expression of VCAM-1 increased with the increase of time, while the expression of E-selectin decreased immediately after the peak of E-selectin expression at 6 h. Based on this, the E-selectin/VCAM-1 double-targeted liposomes were constructed and showed the highest endothelial cell binding rate at 6 and 24 h after stimulated with inflammatory factors, respectively (Gunawan et al., 2011). Therefore, during the design of E-selectin targeted drug delivery system, it is of great significance to construct an effective drug delivery system for targeting the dynamic expression of selectin molecules on the surface of endothelial cells for therapeutic purposes. This, to some extent, poses a challenge to the dynamic monitoring on the expression of selectin molecules on endothelial cells under corresponding pathological conditions.

In addition to synergistic targeted effect, E-selectin targeted strategy is an excellent and promising adjuvant therapy. E-selectin was demonstrated to play a crucial role in cancer cell trafficking, stem-like properties and therapy resistance. A specific E-selectin antagonist, uproleselan, can disrupts the tumor microenvironment by affecting metastasis-extravasation and adhesion (Muz et al., 2021). Additionally, it promoted cancer cells into the circulation and made them more susceptible to chemotherapy (Muz et al., 2021).

## Endothelial cell targeting strategies for respiratory diseases

Pulmonary VECs are the main damaged cells in the occurrence and development of a variety of respiratory diseases. Pulmonary VECs-targeted therapy is important for various respiratory diseases, such as acute lung injury/acute respiratory distress syndrome, pulmonary embolism and pulmonary hypertension.

### Acute lung injury

ALI is a critical case of acute hypoxic respiratory failure resulting from diffuse alveolar-capillary endothelial injury, which can progress to acute respiratory distress syndrome (ARDS). Its mortality rate is up to 30%–40%, thus it could seriously threatening human life and health (Matthay and Zemans, 2011). At present, the treatment for ALI is still based on several supportive options, such as low tidal volume ventilation, positive end-expiratory pressure, pulmonary fluid management, and antimicrobial therapy. Several medications have also been used for ALI, such as vasodilator inhalant (Nitric Oxide, Prostacyclin, etc.), glucocorticoid, ketoconazole, and antioxidants, but the mortality rate for ALI remains high.

**FIGURE 3:**
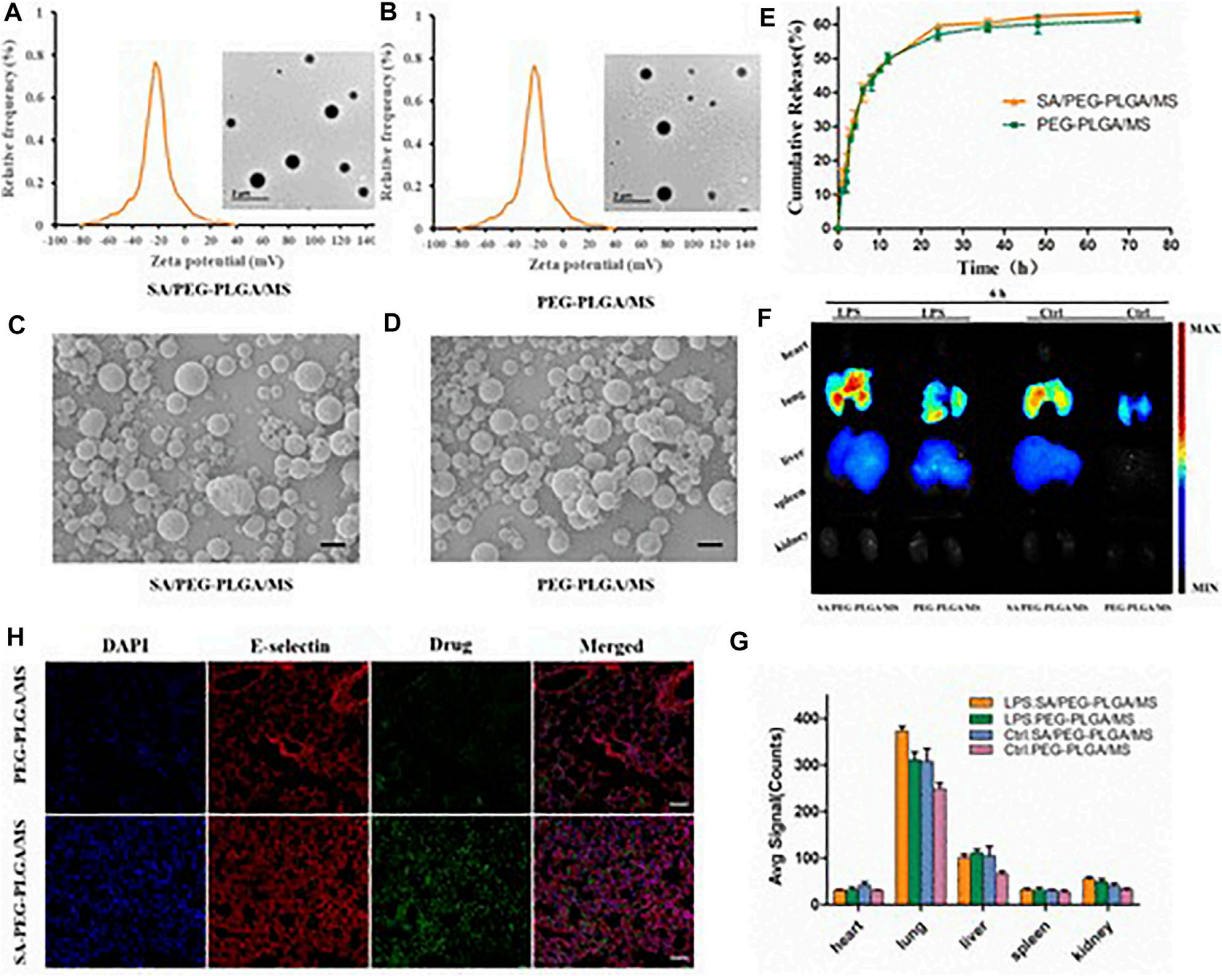
Sialic acid modified microsphere fabrication and characterization. Zeta potential of sialic acid modified PEG-PLGA microspheres **(A)**, PEG-PLGA microspheres **(B)**, obtained by DLS, and the inserted images were obtained by TEM (scale bar = 1 μM). The morphology of SA/PEG-PLGA/MS **(C)** and PEG-PLGA/MS **(D)** were observed by SEM (scale bar = 1 μM). **(E)** *In vitro* release manner of Cur-TPP from SA/PEG-PLGA/MS and PEG-PLGA/MS. **(F)** Bio-distribution of SA/PEG-PLGA/MS *in vivo*. The fluorescence images of excised organs from mice treated with LPS or not. **(G)** The quantitative analysis of fluorescence intensity in heart, lung, liver, spleen and kidney of Figure 2F. (*n* = 3) **(H)** Imaging of E-selectin receptors stained with a fluorescent E-selectin antibody with Cur-TPP loaded microspheres, and nuclei were stained with DAPI. (scale bar = 50 μm) (Jin et al., 2019). It was permitted.

The damage to pulmonary VECs is an important cause of ALI (Craig et al., 2011). The dysfunction of pulmonary VECs can promote the infiltration of inflammatory cells in lung, induce inflammatory cascade reaction, and further damage lung epithelium and endothelium, which form a vicious circle of promoting the imbalanced state between inflammation and anti-inflammation. The design of pulmonary VECs-targeted nanocarriers for the treatment of ALI is of great significance, which can effectively promote drug accumulation in lung and reverse the pathological changes of pulmonary VECs. Inactive NLCs, consisting of phospholipids/triglyceride, were used in HCl-induced ALI mice. NLCs showed good endothelial-protective effects, as reflected by the improved pulmonary microvascular protein leak, airspace inflammatory cells and thrombin proteolytic activity (Kardara et al., 2013). Ferrer et al. (2014) constructed an ICAM-1 mediated PVEC targeting drug delivery system loaded with dexamethasone. The iodine 125-labeled PVEC control group had significant lung targeting advantages and had a good inhibitory effect on the overexpression of pro-inflammatory factors in pathological conditions (Ferrer et al., 2014). In ALI, the increase of macrophages in the alveoli leads to the expression of pro-inflammatory proteins and certain cytokines, and increases chemokines. Therefore, siRNA may be a special therapeutic approach for ALI. A highly efficient siRNA delivery vector targeting tumor necrosis factor was used to investigate its role *in vivo*. Comparison of two different types of phosphate-based dendritic molecules (Pyrrolidinium, morpholinium) showed that the former exhibited stronger siRNA binding, better cellular uptake, and enhanced TNF-α silencing *in vitro* than non-complex siRNA after LPS stimulation. After administration, anti-TNF-α siRNA was targeted to macrophages stimulated by lipopolysaccharide, producing strong anti-inflammatory effects (Bohr et al., 2017). Lung-targeted nanoparticles (ICAM-1/NLC/Pro/Ang), including simvastatin nanolipid carriers conjured with anti-ICAM-1 antibodies, Protamine and Angiopoietin-1, which could not only deliver simvastatin and ANG-1 genes, but also has good distribution and DNA transfer characteristics. *In vitro*, there are better cell uptake and transfection efficiency in EAHY926 cells. After intravenous administration *in vivo*, the TNF-α and IL-6 levels in lung were significantly decreased, the cell infiltration level and tissue structure of LPS-stimulated mice were improved (Jiang et al., 2019). Lung targeting microspheres (MS), sialic acid (SA) modified, loaded with triphenyl phosphonic cation (TPP) modified curcumin (CUR-TPP), could specifically target mitochondria and increase antioxidant capacity. SA could effectively target E-selectin in inflammatory tissues, so it showed good targeting characteristics in ALI mouse models. Compared with the control group, the SA/CUR-TPP/MS group reduced intracellular ROS production, increased mitochondrial membrane potential, and reduced the proportion of apoptosis. The therapeutic effect of SA/CUR-TPP/MS on ALI mouse models was also demonstrated by the generation of oxidative stress and the expression of pro-inflammatory cytokines (Figure 3) (Jin et al., 2019). In addition, studies on lung injury caused by airway smoke inhalation showed that solid lipid nanoparticles (SLN) could minimize oxidative stress and histological damage caused by smoke inhalation in rodents (Shrestha et al., 2012).

### Pulmonary embolism

Pulmonary embolism (PE) is a common clinical pathological syndrome caused by the blocking of the pulmonary artery system by various emboli, which include pulmonary thromboembolism, amniotic fluid embolism, air embolism, and fat embolism. It is a life-threatening condition in which blood clots form in the blood vessels and block or interfere with blood flow. PE has a high morbidity rate, misdiagnosis rate and mortality rate, so it is an acute disease that seriously threatens human life and health. Anticoagulation, thrombolytic therapy and ventilation improvement are the traditional treatment methods for PE. Among them, the thrombolytic action of fibrinolytic solvent such as tenepase, streptokinase, urokinase and recombinant tissue plasminogen activator (tPA) can promote the effective treatment of acute pulmonary embolism. However, such fibrinolsolvent has the side effect of causing internal bleeding, and its poor pharmacokinetics require high-dose drug intervention to some extent, which further increases the risk of bleeding side effect of fibrinolsolvent drugs (Brenner et al., 2015). Targeted delivery of fibrsolvent-based drugs to the pulmonary VECs is expected to achieve safe and effective treatment of acute pulmonary embolism by lowering the dose.

Targeting appropriate markers of VECs, vectors with efficient pulmonary VECs targeting characteristics and long-term sustained-release drugs were constructed to improve the efficacy of thrombolytic therapy by increasing the drug content on endothelial cells. Early studies reported the treatment of PE with ACE antibody-modified tPA, anti-ACE AB/tPA, and the results showed that although anti-ACE AB/tPA had significant distribution, the rapid endocytosis of anti-ACE Ab/tPA by PVEC weakened the thrombolysis of tPA (Muzykantov et al., 1996). Compared with ACE cell markers, ICAM-1 and PECAM-1 mediated VECs-targeted drug delivery systems have better distribution and lower internalization rate by endothelial cells, showing potential preventive and therapeutic advantages for PE. A multifunctional liposome system, consisting of polyethylene glycol, ambient amino-glycine-aspartic acid and tPA, could target the delivery of tPA to the site of thrombosis and improve its drug stability. Moreover, tPA release can be controlled by activating platelet concentration (Huang et al., 2019). The single-chain variable fragment scFV modified low-molecular-weight single-chain urokinase plasminogen activator, scFV/LMW-SCUPA, Compared with the unmodified LMW-SCUPA, scFV/LMW-SCUPA had a significant advantage in lung distribution and dissolved pulmonary microsupposas more effectively, reflecting the potential application of PECAM-1 targeting in PE prevention (Greineder et al., 2013).

Thrombolytic agents (TAs) can promote the conversion of plasminogen to plasminase, break down fibrin clots into soluble fibrin, and dissolve thrombus. But systemic injections can cause nonspecific activation and increase the rate of bleeding. Therefore, the P-selectin targeting nanocarriers could target TAs to the site of thrombosis, increase drug utilization, reduce side effects, and improve the overall efficacy (Hassanpour et al., 2020).

### Pneumonia

Pneumonia refers to inflammation of the lungs, which is a frequent and common disease of the respiratory system. It occurs mostly in the young and the elderly, or people with immune deficiency and poor immune system. According to the investigation of the World Health Organization, pneumonia had a relatively high mortality rate in acute respiratory infections. Based on different pathogenic factors, pneumonia can be divided into allergic pneumonia, physical and chemical pneumonia and infectious pneumonia, with the main sources of infection including bacteria, fungi, and viruses (Koenig and Truwit, 2006). In terms of pathogenesis, pneumonia is a disease in which foreign pathogens interact with the lungs to trigger an inflammatory response that causes the bronchioles and alveoli to fill with fluid, subsequently resulting in lung tissue to collapse and making it difficult to breathe (Muhammad et al., 2021).

**FIGURE 4:**
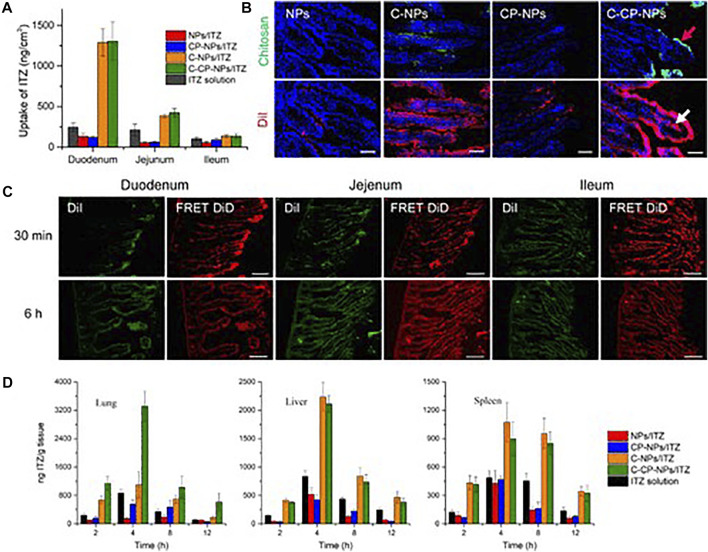
Enhanced intestinal absorption of encapsulated drugs and *in vivo* targeting efficacy of C-CP-NPs. **(A)** *In vivo* intestinal absorption of ITZ of different formulations. **(B)** Fluorescence images showing localization of chitosan and NPs in duodenum, where chitosan was labeled with FITC (green) and NPs were labeled with DiI (red). **(C)** Fluorescence images of DiI channel (green) and FRET DiD channel (red) at 30 min and 6 h post-oral administration. Scale bars, 100 µm. **(D)** Tissue distribution of ITZ in major organs of C. Neoformans-infected mice after oral administration of 40 mg kg^−1^ NPs loaded with ITZ or marketed drug solutions. Data are shown as mean ± SD (*n* ≥ 3) (Tang et al., 2018). It was permitted.

There are several effective drug therapies for pneumonia, including many types of antibiotics and vaccines, which can reduce the severity of the disease by suppressing the pathogen. However, the immune response caused by inflammation of lung tissue cannot be treated in time, which may lead to the miss of optimal treatment time, resulting in more serious illness. Inflammation-regulating nanoparticles can be loaded with drugs, targeted for transport and released into the site of inflammation, thus effectively suppressing immune cells and reducing inflammation (Muhammad et al., 2021). With poly (lactic co-glycolic acid) as vectors, chitosan binding peptides screened by phages were coupled to load itraconazole, which could form oral administration nanoparticles, C-CP-NPS/ITZ (Figure 4) (Tang et al., 2018). The non-covalent interaction between chitosan and polypeptide enabled the drug carrier to respond to the physiological changes from the intestine to the circulation, successfully realizing the two-step targeting function. The average particle size of CP-NPS/ITZ measured by dynamic light scattering (DLS) is 112 nm, while that of C-CP-NPS/ITZ is 136 nm. The potential of CP-NPS/ITZ was 6.1 mV, which increased to 21.5 mV after binding with chitosan. The minimum inhibitory concentration (MIC) of NPs/ITZ was 100 g ml^−1^. However, chitosan combined with peptide modification significantly improved the antifungal effect by an order of magnitude (MIC = 10 g ml^−1^). Compared with unbound NPs, the drug absorption levels of chitosan-bound NPs showed significantly higher values. In addition, nanoparticles carry anti-inflammatory drugs, peptides and proteins that have been used to target macrophages, dendritic cells and B cells to suppress inflammation.

In recent years, COVID-19 caused by SARS-CoV-2 has become a globally contagious pneumonia. Coronaviruses can travel through the nasal mucosa, olfactory fibers, or blood to the central nervous system, where they infect endothelial cells, pericytes, and even neurons, causing protein-rich edema and fibrin fragments in the lungs, with a fatality rate of up to 70% (Khan et al., 2020). ACE is the SARS-CoV-2 receptor, so targeted inhibition of ACE or the use of angiotensin receptor blockers can have a positive effect on the disease (Okada et al., 2021). In addition, vascular endothelial glycocalyx plays an important role in vascular endothelial injury, thrombosis and inflammation. Since systemic neovascularization is caused by inflammation and vascular endothelial injury, the prevention and treatment of endothelium glycocalyx may have positive therapeutic effects (Miners et al., 2020).

## Conclusion

There are many respiratory diseases, most of which will cause symptoms such as poor breathing and asthma. In serious cases, they will also cause chain reactions in the heart, stomach and other organs. The development of many drugs for respiratory diseases is limited by their hydrophobicity or non-specific distribution. Pulmonary VECs targeting opens up a new way for the effective treatment of respiratory system diseases, especially vascular lung diseases. According to the corresponding diseases and the condition of the body, the design of the appropriate targeted drug delivery systems of pulmonary VECs can promote the effective accumulation of drugs in the lungs, making it possible to realize the effective prevention, diagnosis and treatment of various respiratory diseases. Here, we summarized pulmonary VECs markers and analyzed their application, which can help better understand the whole lung targeting system. With the further study on the expression characteristics and functional mechanism of endothelial cell markers, the further optimization of drug delivery system design and the perfection of *in vivo* safety monitoring, we believe that pulmonary VECs targeting will make a significant contribution to the treatment of human respiratory diseases.
